# BK virus-associated nephropathy in a lung transplant patient: case report and literature review

**DOI:** 10.1186/s12879-020-05292-0

**Published:** 2020-08-14

**Authors:** Thomas Crowhurst, James Nolan, Randall Faull, Mark Holmes, Chien-Li Holmes-Liew

**Affiliations:** 1 Discipline of Medicine, University of Adelaide, Adelaide, SA 5000 Australia; 2 SA Lung Transplant Service, Royal Adelaide Hospital, Central Adelaide Local Health Network, 1 Port Road, Adelaide, SA 5000 Australia; 3 SA Pathology, Royal Adelaide Hospital, Central Adelaide Local Health Network, 1 Port Road, Adelaide, SA 5000 Australia; 4 Central Northern Adelaide Renal and Transplantation Service, Royal Adelaide Hospital, Central Adelaide Local Health Network, 1 Port Road, Adelaide, SA 5000 Australia

**Keywords:** BK virus, Nephropathy, End-stage renal failure, Lung transplantation, Case report

## Abstract

**Background:**

BK virus-associated nephropathy (BKVAN) is a relatively common cause of renal dysfunction in the first six months after renal transplantation. It arises from reactivation of the latent and usually harmless BK virus (BK virus) due to immunosuppression and other factors including some that are unique to renal transplantation such as allograft injury. BKVAN is much rarer in non-renal solid organ transplantation, where data regarding diagnosis and management are extremely limited.

**Case presentation:**

We report a case of a 58-year-old man found to have worsening renal dysfunction nine months after bilateral sequential lung transplantation for chronic obstructive pulmonary disease (COPD). He had required methylprednisolone for acute allograft rejection but achieved good graft function. Urine microscopy and culture and renal ultrasound were normal. BK virus PCR was positive at high levels in urine and blood. Renal biopsy subsequently confirmed BKVAN. The patient progressed to end-stage renal failure requiring haemodialysis despite reduction in immunosuppression, including switching mycophenolate for everolimus, and the administration of intravenous immunoglobulin (IVIG).

**Conclusions:**

This very rare case highlights the challenges presented by BK virus in the non-renal solid organ transplant population. Diagnosis can be difficult, especially given the heterogeneity with which BKV disease has been reported to present in such patients, and the optimal approach to management is unknown. Balancing reduction in immunosuppression against prevention of allograft rejection is delicate. Improved therapeutic options are clearly required.

## Background

BK virus (BKV) is a non-enveloped double-stranded DNA polyomavirus. It resides in renal tubular and uroepithelial cells, causing no sequelae in immunocompetent individuals [1]. Primary infection occurs in childhood and as many as 80% of adults demonstrate serological evidence of exposure [2]. Intermittent viral replication manifests as asymptomatic viruria in 7–15% of healthy people [3]. Immunocompromise can enable significant reactivation whereby BKV can progress from viruria to viraemia and cause end-organ disease, usually BKV-associated nephropathy (BKVAN) in renal transplant recipients and haemorrhagic cystitis in haematopoietic stem cell transplant recipients. Reports of BKVAN in other states of immunocompromise, especially after lung transplantation, are rare.

BKV viruria occurs in 25–30% of renal transplant recipients and 11–13% develop viraemia, with 1–10% progressing to BKVAN usually by 6 months post-transplant [4, 5]. The key risk for BKVAN is degree of immunosuppression; other factors include ABO incompatibility, tacrolimus-containing regimen, donor-positive to recipient-negative serostatus, extremes of age and male sex [6–9]. Higher pre-transplant levels of neutralising anti-BKV antibodies may reduce the risk of BKVAN [10]. The rarity with which the disease affects native kidneys highlights unique characteristics of renal transplantation that engender a more permissible environment for reactivation; these include allograft injury from surgery and ischaemia, rejection, HLA mismatch impacting host immune activity in the allograft, and others [11, 12].

Screening for BKVAN is recommended after renal transplantation and involves monitoring for BKV viraemia via quantitative polymerase chain reaction (PCR), given viraemia is a reliable precursor to BKVAN [13]. Persistent or significant viraemia is pre-emptively managed with reduction of immunosuppression before allograft dysfunction arises [5]. Screening is not recommended in other solid organ transplantation. Electron microscopy for cast-like urinary polyomavirus aggregates (‘Haufen’) is highly sensitive and specific for BKVAN, and may be particularly useful in paediatric patients where biopsy is difficult [14, 15]. In renal transplantation, biopsy is reserved for when there is diagnostic uncertainty or when reduction of immunosuppression has not led to improvement in renal dysfunction or viraemia. Histological findings in BKVAN share similarities with other viral nephropathies and include intranuclear viral inclusions, tubular damage, and mononuclear or polymorphonuclear infiltrates in affected areas; confirmation occurs via positive immunohistochemistry (IHC) for simian virus 40 (SV40) large T antigen, which cross-reacts with BKV and other polyomaviruses like JC virus (JCV) [16]. The Banff Working Group Classification of Definitive Polyomavirus Nephropathy was published in 2018 on the basis of a large retrospective analysis of proven polyomavirus nephropathy cases; it seeks to provide a consensus morphologic grading scheme that reflects important clinical parameters including presentation at diagnosis, renal function after index biopsy, and future graft failure [6].

Reduction in immunosuppression is the only proven strategy for management of BKVAN, however there is no universally agreed approach. There is some evidence mTOR inhibitors, compared with calcineurin inhibitors and mycophenolate, could enable superior control of BKVAN without increasing risk of rejection [17, 18]. Other therapies such as intravenous immunoglobulin (IVIG), leflunomide and cidofovir are unproven but occasionally employed. Quinolone antibiotics have been shown to provide no benefit while increasing antimicrobial resistance [19]. Future treatments may include brincidofovir and allogeneic polyomavirus-specific T cell therapy [20, 21].

## Case presentation

A 58-year-old man was noted to have 3 months of progressive renal dysfunction at routine outpatient follow-up for his bilateral sequential lung transplant, which was performed 9 months earlier for severe chronic obstructive pulmonary disease (COPD). Methylprednisolone alone was used for induction. He was found to have acute rejection (A1B0) on surveillance bronchoscopy at 2 months post-transplant and was managed with pulse methylprednisolone with good results. His immunosuppressive regimen consisted of prednisolone, tacrolimus and mycophenolate. Before the onset of renal dysfunction, his prednisolone dose was 10 mg daily and mycophenolate dose was 500 mg twice daily. Median tacrolimus trough level from months three to six post-transplant was 11.2 μg/L (interquartile range [IQR]: 8.6–13.7 μg/L). He had not experienced further rejection and his graft function was good with a forced expiratory volume in 1 s (FEV1) of 2.44 l (82% predicted and 91% of post-transplant baseline).

Other post-transplant issues included (a) positive galactomannan on three-month surveillance bronchoscopy without invasion which was managed with itraconazole, (b) respiratory syncytial virus and rotavirus at 3 months post-transplant, (c) steroid-induced diabetes mellitus which settled with reduction in prednisolone, (d) hypertension controlled with angiotensin receptor blockade, (e) persistent small left effusion drained at 10 months post-transplant, (f) asymptomatic cytolomegalovirus (CMV) viraemia detected at 10 months post-transplant and treated with valganciclovir, (g) iron deficiency anaemia due to dysplastic colonic polyps which were removed, (h) cataracts and (i) benign squamous papilloma affecting the right main bronchus anastomosis which was detected at 15 months post-transplant due to declining allograft function and which improved with balloon dilatation. Histological assessment did not demonstrate a viral aetiology for the bronchial squamous papilloma, however specific testing for human papilloma virus was not performed.

Background medical problems prior to transplant included coeliac disease, central sleep apnoea managed with oxygen, osteoporosis and hypercholesterolaemia. Pharmacological therapy at the time of detection of renal dysfunction, in addition to his immunosuppression, included: azithromycin, pantoprazole, candesartan, atorvastatin, calcium citrate, vitamin D, annual zoledronic acid, magnesium supplementation plus prophylactic itraconazole, trimethoprim/sulfamethoxazole and valganciclovir.

The patient had a pre-transplant glomerular filtration rate (GFR) of 85 ml/min/1.73m^2^ and a stable baseline of 70 ml/min/1.73m^2^ at 6 months post-transplant; this relatively mild stepwise decline in renal function was attributed to multifactorial peri-operative renal injury, as is seen very often after lung transplantation. His renal function then progressively deteriorated over 3 months to reach a GFR of 35 ml/min/1.73m^2^ at 9 months post-transplant despite reduced tacrolimus trough levels over this period (median: 5.2 μg/L | IQR: 4.5–5.9 μg/L). Urinary microscopy and culture analyses were bland on two separate occasions over 3 days with no haematuria, pyuria, decoy cells or casts. Ultrasound of the renal tract performed at the same time was normal with no evidence of obstruction and normal renal size. Given the absence of another identifiable cause and the immunosuppression, PCR for BKV was performed on samples collected at the same time as those for urinary microscopy and culture; the PCR was positive at > 10 million copies/ml in urine and 358,000 copies/ml in blood. Nephrology input was sought. Mycophenolate was ceased, target tacrolimus level was reduced to 5 μg/L, everolimus was commenced with a target level of 2.5 to 3 μg/L and a biopsy was planned.

**Fig. 1:**
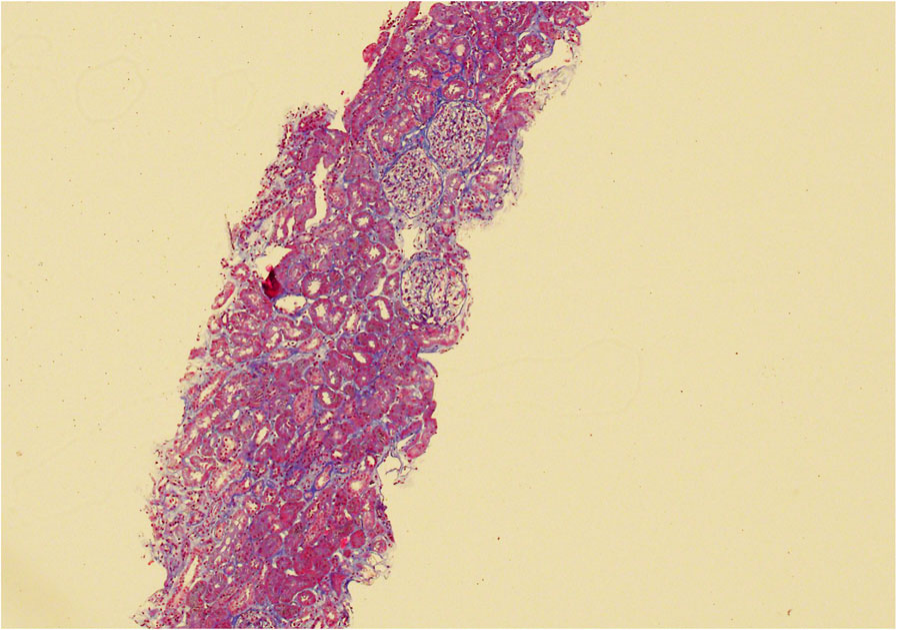
Histological section of renal biopsy at 100 times magnification with trichrome stain showing no significant chronic damage to the renal cortical parenchyma.

**Fig. 2:**
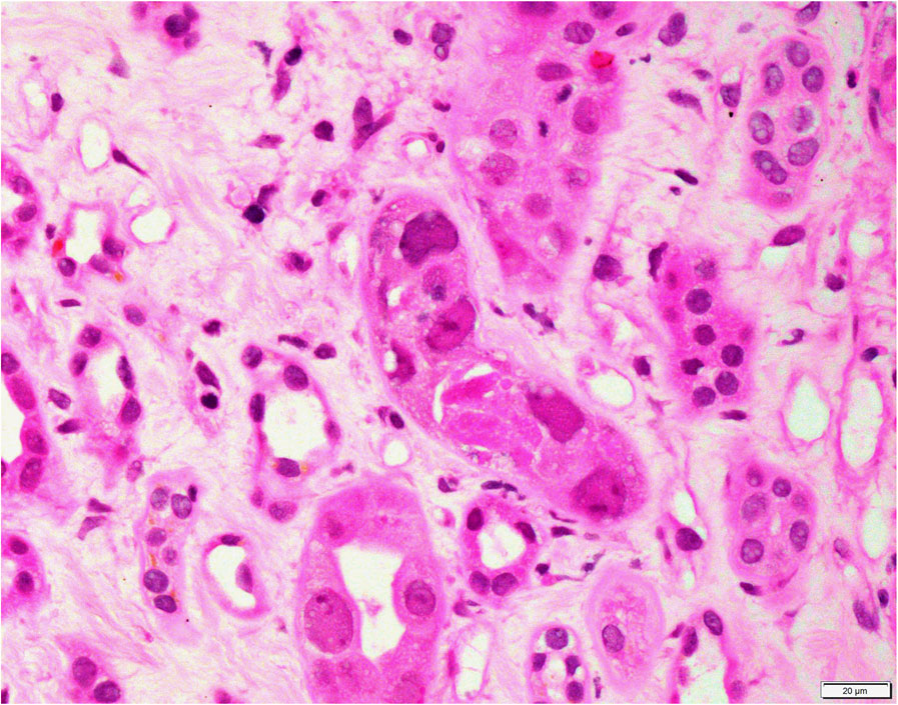
Histological section of renal biopsy at 600 times magnification showing viral cytopathic effect

**Fig. 3:**
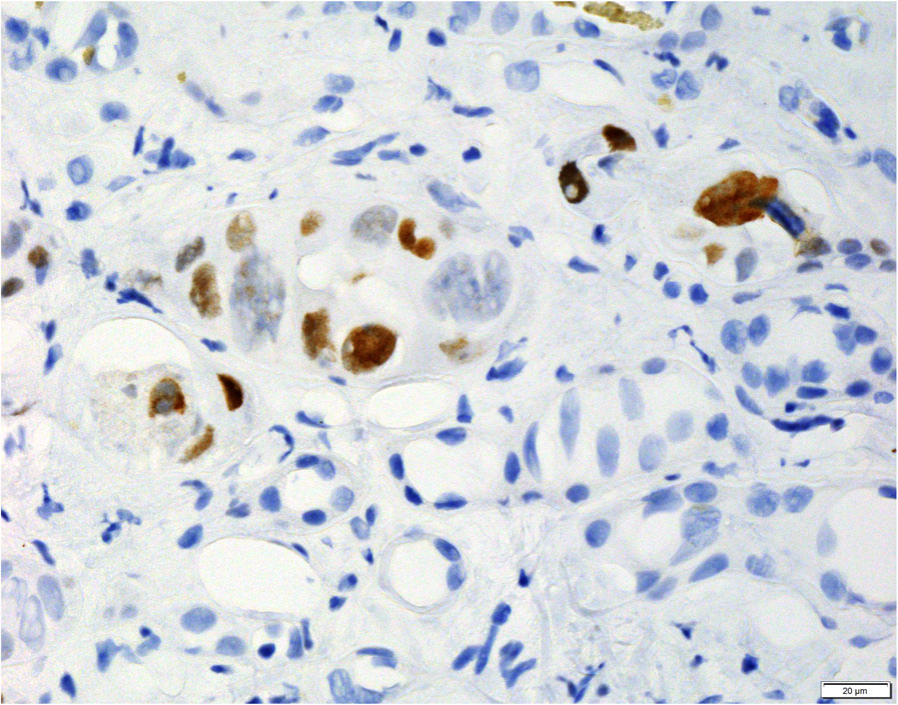
Immunohistochemical stain with antibody to SV40 at 600 times magnification showing positively staining nuclei of tubular epithelial cells

**Fig. 4:**
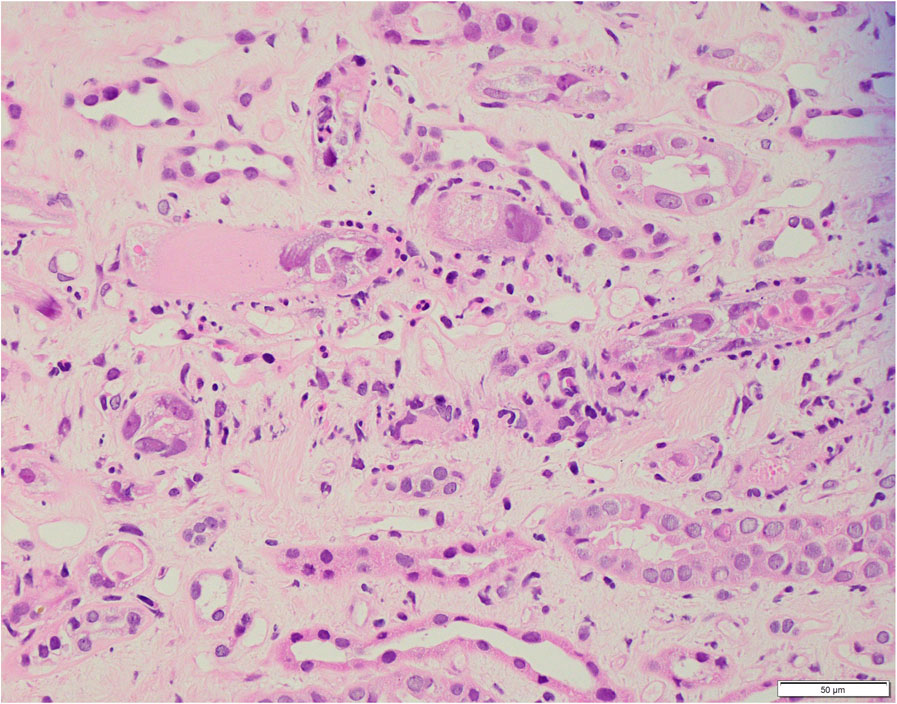
Histological section of renal biopsy at 400 times magnification showing interstitial oedema, tubulitis and viral cytopathic effect

A renal biopsy was performed 3 weeks after the positive BKV PCR result (Figs. 1, 2, 3 and 4). The biopsy comprised a formalin fixed 9 mm core of renal tissue including 50% each of cortex and medulla. Routine renal biopsy haematoxylin and eosin histology sections were produced and routine histochemical stains performed. There were up to 7 viable glomeruli per section and no obsolescent forms. The glomeruli showed no morphologic abnormality. There was mild cortical fibrosis, less than 10% of cortical area, with Banff ci1 assigned. There was no cortical inflammation. The biopsy included a portion of an arterial vessel with mild to moderate arteriosclerosis. Arterioles were normal. In medullary tubules adjacent to the cortico-medullary junction were cells with abnormal nuclei consistent with viral cytopathic effect; nuclear inclusions were seen along with lymphocytic tubulitis and sloughed epithelial cells. In this region, IHC with antibody to SV40 was positive in the nuclei of epithelial cells. The involved tubules were quantified according to the method described by Nickeleit et al. [6]. This rendered a count of 2.5% and a *pvl* score of 2 (1 to 10%). The combination of Banff ci1 and *pvl* 2 produced an overall classification of polyomavirus nephropathy class 2. Tissue was not submitted by the clinician for immunofluorescence. Electron microscopy of tissue received in glutaraldehyde showed glomeruli with ischaemic alterations only; no viral particles were seen in the tissue.

**Fig. 5:**
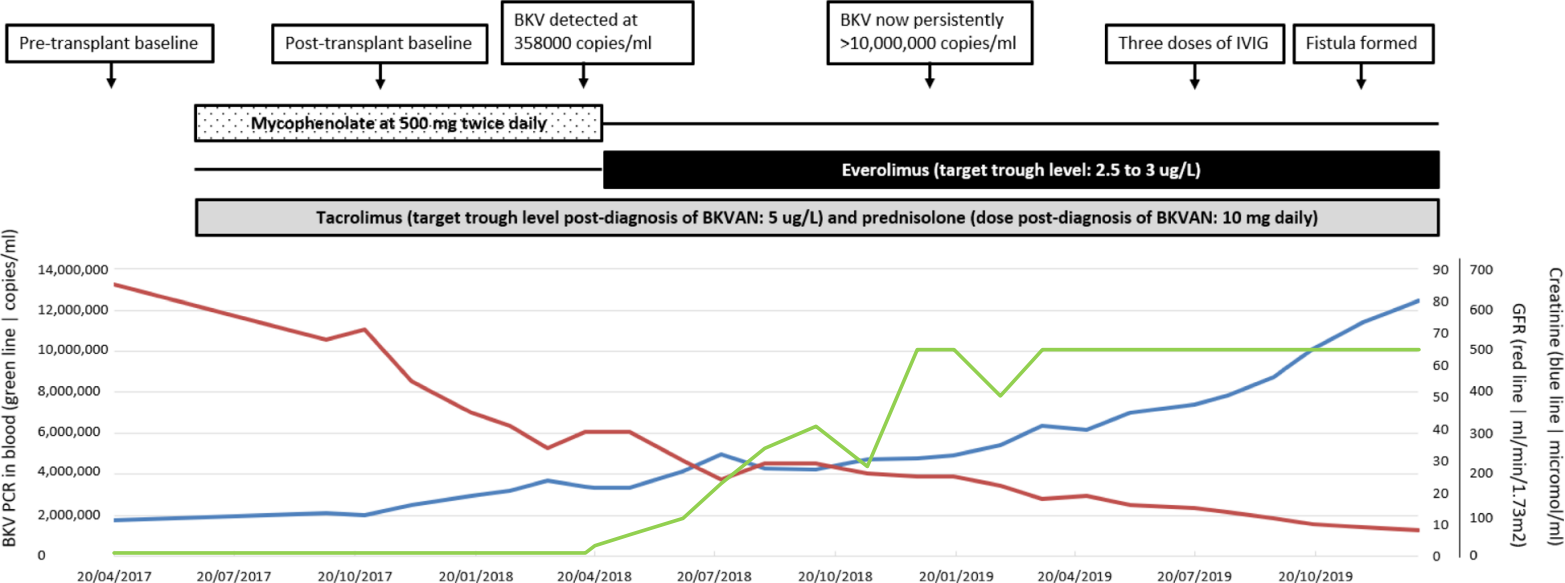
Graphic depiction of patient progress with time on the X-axis and viral load in blood (green line) on the left Y-axis, with creatinine (blue line) and glomerular filtration rate on the right Y-axis (red line). BKV: BK virus. IVIG: intravenous immunoglobulin. BKVAN: BK virus-associated nephropathy. GFR: glomerular filtration rate

The combination of the clinical, virological and histological findings allowed a definitive diagnosis of polyomavirus nephropathy due to BKV. Decline in renal function continued despite reduction in immunosuppression. The quantitative BKV PCR in blood climbed over the ensuing 8 months to reach and remain > 10,000,000 copies/ml. Three doses of IVIG were given over 2 months but this was then ceased due to an absence of effect. Patient progress is summarised in Fig. 5. He was regularly reviewed by nephrology and, at 20 months post-diagnosis of BKVAN and 29 months post-transplant, he had an arteriovenous fistula formed for planned commencement of haemodialysis.

## Discussion and conclusions

This case is the eighth reported instance of BKVAN after lung transplantation, the seventh where a confirmatory biopsy has been performed and the first in which the Banff classification for polyomavirus nephropathy has been applied [22–28]. Reported cases are summarised in Table 1. Our case is worthy of discussion because of its rarity and because it highlights the dilemmas associated with organ-threatening infections in immunosuppressed transplant recipients.

**Table 1:** Summary of all reported cases of BK virus-associated nephropathy in lung transplant recipients including the present case

Author and year	Age at transplant Gender	Indication for transplant	Onset of renal dysfunction (months post-transplant) Creatinine at disease onset (μmol/l)	BKV PCR in urine and blood at diagnosis	Biopsy	Management	Outcome
Schwarz et al. 2005 [15]	38 years Male	Pulmonary fibrosis due to chemotherapy for seminoma	15 months 227 μmol/l	Urine: + > 100,000,000 Geq/ml Blood: + 117,500 Geq/ml	Positive	Immunosuppression not reduced due to recent rejection, hence cidofovir used, then leflunomide in place of cidofovir due to improved biopsy results	Repeat renal biopsy performed three months after original diagnostic biopsy and one month after a course of cidofovir showed absence of BKVAN changes (with BKV PCR in blood also showing significant reduction) with further subsequent improvement on leflunomide, however renal function nonetheless deteriorated and RRT was required
Egli et al. 2010 [16]	67 years Female	COPD	63 months 183 μmol/l	Urine: + > 100,000,000 Geq/ml Blood: + 71,000 Geq/ml	Positive	↓ immunosuppression, leflunomide (but was ceased at 3 months for diarrhoea / anaemia)	Stabilisation of creatinine (peak was at 237 μmol/l improving to 190 μmol/l) and undetectable BKV PCR in blood (still positive in urine) at 1 year post-diagnosis
Dufek et al. 2013 [17]	8 years Male	Bronchiolitis obliterans	12 months N/A	Urine: > 100,000,000 Geq/ml Blood: + > 100,000,000 Geq/ml	Positive	Haemodialysis, switch of mycophenolate for everolimus, ↓ tacrolimus and prednisolone, intravenous cidofovir	Development of rapidly progressive, ultimately fatal, collecting duct carcinoma with strong positivity for SV40 antibody staining in the nuclei of tumour cells
Sharma et al. 2013 [18]	30 years Male	Cystic fibrosis	24 months 195 μmol/l	Urine: N/A Blood: + 3,500,000 Geq/ml	Positive	Leflunomide commenced and mycophenolate ceased	Increase then stabilisation of creatinine at 274 μmol/l at 20 months post-diagnosis
Vigil et al. 2016 [19]	70 years Male	IPF	24 months 265 μmol/l	Urine: N/A Blood: + 10,000,000 Geq/ml	Positive	Mycophenolate was ceased, tacrolimus and prednisolone continued, leflunomide started and three doses of IVIG given	Improvement in BKV PCR in blood and stabilisation of creatinine at 250 μmol/l
Kuppachi et al. 2017 [20]	63 years Male	COPD	24 months 230 μmol/l	Urine: N/A Blood: + 87,900 Geq/ml	Positive	Azathioprine ceased, ↓ tacrolimus, leflunomide commenced	Initial good response with reduction in BKV PCR in blood and stabilisation of renal function at 265 μmol/l, but then was found to have locally-advanced prostate carcinoma and separate metastatic urothelial carcinoma (two separate primary malignancies) which rapidly advanced in parallel with drastic increases in BKV PCR counts
Okumura et al. 2019 [21]	30 years / 44 years Female	LAM/relapsed LAM	3 months 66 μmol/l	Urine: + > 100,000,000 Geq/ml Blood: + 800 Geq/ml	N/A	↓ immunosuppression to standard maintenance levels post-transplant, and addition of sirolimus at six months post-transplant	Gradual improvement in renal function and reduction in BKV PCR in blood, with levels falling to undetectable levels at 5 months post-diagnosis
Present case 2020	57 years Male	COPD	9 months 184 μmol/l	Urine: + > 10,000,000 Geq/ml Blood: + 358,000 Geq/ml	Positive	↓ immunosuppression, change of mycophenolate to everolimus, and then IVIG when renal function deteriorated further	Gradual deterioration in renal function despite these measures, requiring fistula formation for the planned commencement of haemodialysis

Legend for Table 1: table summarising the eight reported cases of BKVAN in lung transplant recipients. *BKVAN* BK virus-associated nephropathy. *BKV* BK virus. *PCR* Polymerase chain reaction. *Geq/ml* Genome equivalents per millilitre. *RRT* Renal replacement therapy. *COPD* Chronic obstructive pulmonary disease. *N/A* Information not available from publication. *SV40* Simian virus 40. *IPF* Idiopathic pulmonary fibrosis. *IVIG* Intravenous immunoglobulin. *LAM* Lymphangioleiomyomatosis

Our case shares some similarities with those previously reported. Unlike renal transplant where BKVAN usually arises within 6 months, our case replicates the pattern of somewhat later presentation observed in earlier reports of BKVAN after lung transplant; median time to presentation across the eight reported cases is 19.5 months with IQR of 11–24 months. As with three of the other seven cases, our patient was more immunosuppressed than would be usual following lung transplantation due to the additional methylprednisolone for his acute rejection; in two of the three other cases the reason was also treatment for acute rejection whereas in the third it was neutropenia due to trimethoprim/sulfamethoxazole [22–24]. Higher levels of immunosuppression may have led to BKVAN in these patients, but this leaves the question as to what caused disease in the other four cases where immunosuppression was at routine levels. Urinary microscopy was bland in our patient, as it was in two of the other three cases for which results have been reported; only Okumura et al. have demonstrated decoy cells associated with BKVAN in a lung transplant patient [25, 26, 28].

Our case is only the second reported instance where BKVAN has progressed to end-stage renal failure despite reduction in immunosuppression. This occurred in parallel with persistent extremely high BKV PCR levels measured in blood. There are no obvious factors that explain the poor outcome of our patient compared with the previously reported cases, however analysis is challenging given the paucity of data. BKV appears to have heterogenous manifestations in lung transplant recipients, noting the development of aggressive urothelial carcinoma in two of the reported cases almost certainly due to viral oncogenic effect, and with another report by Elidemir and colleagues describing haemorrhagic cystitis from BKV in a paediatric lung transplant recipient [24, 27, 29].

Some studies have attempted to determine whether non-renal solid organ transplant recipients should undergo to a similar screening regime to detect BK viraemia and prevent BKVAN. Barton and colleagues performed a prospective cross-sectional study of consecutive non-renal solid organ transplant recipients with unexplained chronic renal dysfunction of at least 3 months duration, with 65% of their 34 subjects being lung transplant recipients [30]. None of the patients had viraemia and only 15% had viruria; they associated viruria with mycophenolate use and a history of CMV disease, but GFR was similar in those with and without viruria. Thomas et al. undertook a prospective study of 50 lung transplant recipients, analysing urine and blood samples over a 17-month period for BKV but also JCV and SV40 [31]. All blood samples were negative. Urine was positive for BKV on at least one occasion in 32% of patients, while JCV and SV40 were detected at least once in 24 and 12% of subjects respectively. Doucette et al. performed a 9-month study of BKV in 60 patients with non-renal solid organ transplants, with 47% being lung transplant recipients; viruria was found in 15% but viraemia was not detected and there was no significant difference in GFR between those with and without viruria [32]. These studies seem to suggest that although polyomavirus viruria is common and harmless in lung transplant recipients, viraemia and BKVAN appear to be rare events.

The apparent clinical insignificance of BKV viruria has, however, been questioned by another more recent study by Thomas and colleagues [33]. They followed 99 lung transplant patients for 4.5 years with urine samples tested for BKV, JCV and SV40. Polyomavirus viruria occurred at least once in 66% of cases (BKV 42% | JCV 28% | SV40 7%) and was positively associated with COPD but, surprisingly, negatively associated with acute rejection. Patients with viruria did not have significantly different renal function overall, however transient dysfunction was temporally associated with viruria episodes. Importantly, BKV viruria was associated with reduced survival however the magnitude of this effect was not reported; viruria was associated with chronic lung allograft dysfunction (CLAD) as a cause of death, with 26% of those with viruria dying of CLAD versus 10% of those without (*p* = 0.047). Causation is unproven and multiple other factors may explain these findings, but this study raises questions.

There are no data beyond case reports to indicate the reliability of BKV viraemia as a surrogate marker for risk of BKVAN in lung transplant recipients. The evidence in renal transplant recipients is strong [13]. Razonable and colleagues conducted a retrospective analysis of samples collected during a longitudinal study of CMV in solid organ transplant recipients including renal transplants but not including lung transplants [34]. BKV viraemia was found in 26% of renal, 6.7% of heart and 4.1% of liver transplants in the first year post-transplant, at a median of 100 days. All three positive cardiac transplant cases and one of five positive liver cases developed BKV viraemia after treatment for acute allograft rejection. None of the non-renal solid organ transplant patients with BKV viraemia developed renal dysfunction. Salama et al. found no BKV viraemia and no association between BKV viruria and renal function in 41 liver transplant recipients [35]. Louches et al. conducted a prospective longitudinal study of a consecutive sample of 28 heart transplant patients, finding 21% developed viraemia and 43% developed viruria; two of the five viraemic patients developed renal impairment [36]. Application of these data to lung transplant recipients is difficult. We believe that the optimal approach at this time is to monitor renal function in lung transplant recipients regularly and to test for BKV when there is persistent renal dysfunction of uncertain cause; however, we feel there is insufficient evidence to support routine surveillance for BKV viraemia in all lung transplant recipients. BKV viruria is relatively common in this patient population and does not appear to be sufficiently correlated with end-organ disease to warrant its use as a surveillance measure. Biopsy remains important for many reasons including rare cases of nephropathy from other polyomaviruses [37].

BKV is a rare but important cause of disease in lung transplant recipients with manifestations including BKVAN, haemorrhagic cystitis and urothelial carcinoma. Research could examine factors causing BKV reactivation and disease in these patients; it is interesting to consider whether donor-positive to recipient-negative serostatus might be a risk factor and, if so, whether this would imply BKV transmission via lung transplantation. The optimal approach to diagnosis is unclear and the role of screening requires further investigation. Improved treatments are required and may include allogeneic polyomavirus-specific T cell therapy.

## Data Availability

All relevant data for this case are shared in this manuscript. Further provision of data will not be contemplated due to the priority of patient confidentiality.

## Abbreviations

BKV: BK virus
BKVAN: BK virus-associated nephropathy
PCR: Polymerase chain reaction
IHC: Immunohistochemistry
SV40: Simian virus 40
JCV: JC virus
IVIG: Intravenous immunoglobulin
IQR: Interquartile range
COPD: Chronic obstructive pulmonary disease
FEV1: Forced expiratory volume in 1 s
CMV: Cytolomegalovirus
GFR: Glomerular filtration rate
